# Observational Study of 180° Turning Strategies Using Inertial Measurement Units and Fall Risk in Poststroke Hemiparetic Patients

**DOI:** 10.3389/fneur.2017.00194

**Published:** 2017-05-15

**Authors:** Rémi Pierre-Marie Barrois, Damien Ricard, Laurent Oudre, Leila Tlili, Clément Provost, Aliénor Vienne, Pierre-Paul Vidal, Stéphane Buffat, Alain P. Yelnik

**Affiliations:** ^1^Cognition and Action Group, Cognac-G, CNRS UMR 8257, Université Paris Descartes, Service de Santé des Armées, Paris, France; ^2^Service de Neurologie, Hôpital d’Instruction des Armées de Percy, Service de Santé des Armées, Clamart, France; ^3^École d’application du Val-de-Grâce, Service de Santé des Armée, Paris, France; ^4^Institut Galilée, Université Paris 13, Villetaneuse, France; ^5^PRM Department, GH St Louis Lariboisière F. Widal, AP-HP, Paris Diderot University, Paris, France; ^6^Institut de Recherche Biomédicale des Armées, Bretigny-sur-Orge, France

**Keywords:** stroke, 180° turn, turning strategy, inertial measurement unit, fall

## Abstract

**Objective:**

We analyzed spontaneous 180° turning strategies in poststroke hemiparetic patients by using inertial measurement units (IMUs) and the association of turning strategies with risk of falls.

**Methods:**

We included right paretic (RP) and left paretic (LP) post-stroke patients, and healthy controls (HCs) from a physical and rehabilitation department in France between July 2015 and October 2015. All subjects were right-handed and right-footed for mobilization tasks. Participants were instructed to turn 180° in a self-selected direction after a 10-m walk while wearing three IMUs on their trunk and both feet. We defined three turning patterns based on the number of external steps (pattern I = 1; II = 2–4 steps; and III ≥ 5) and four turning strategies based on the side chosen to turn (healthy or paretic) and the stance limb used during the first step of the turn (healthy or paretic). Falls in the 6 months after measurement were investigated.

**Results:**

We included 17 RP [mean (SD) age 57.5 (9.5) years (range 43–73)], 20 LP patients [mean age 60.7 (8.8) years (range 43–63)], and 15 HCs [mean age 56.7 (16.1) years (range 36–83)]. The LP and RP groups behaved similarly in turning patterns, but 90% of LP patients turned spontaneously to the paretic side versus 59% of RP patients. This difference increased with turning strategies: 85% of LP versus 29% of RP patients used strategy 4 (paretic turn side with paretic limb). Patients using strategy 4 had the highest rate of falls.

**Conclusion:**

We propose to consider spontaneous turning strategies as new indicators to evaluate the risk of fall after stroke. IMU could be routinely used to identify this risk and guide balance rehabilitation programs.

## Introduction

Falling is a common complication in poststroke ambulatory patients with hemiparesis, and depending on the study, it occurs in 14–65% of patients during hospitalization (1, 2) and 37–73% after discharge from a hospital (3, 4). Many fall risk factors such as the stroke location are debated (5–9). Activities at the time of fall commonly involve daily transferring tasks, which suggests that turning and more precisely the stepping pattern during turning could be particularly problematic for poststroke patients [see review in Ref. (10)].

In healthy populations, a 180° turn consists of a complex and varied foot-stepping pattern that leads to a smooth continuous top-down rotation from the head to the trunk (11–16). Two main turning strategies have been identified: the spin turn involves a change in direction toward the side of the stance limb and the step turn involves a change in direction away from the stance limb (12, 13). The spin strategy is less stable because it leads to a reduced base of support (12, 13, 17–19). Clinically, the number of external steps and the turn duration are used to evaluate turning (11). The spontaneous turn direction patients choose is also a good indicator because it demonstrates the strategies they are able to develop.

Poststroke consequences (paresis, hypoesthesia, and visuospatial neglect) cause marked alterations in 180° turning, including increased number of external steps, decreased turn velocity, and altered axial segment coordination (20–22). Right hemispheric stroke presents specific cognitive deficits (anosognosia, somatagnosia, and neglect), which could be involved in fall occurrence (10). These deficits likely affect the choice of turning strategy. To date, turn duration was found the only significant variable differentiating faller from non-faller paretic patients (22). However, turning strategies have not been studied in poststroke survivors. To the best of our knowledge, no study has focused on differences in 180° turns between left paretic (LP) and right paretic (RP) patients. Such an analysis is important to evaluate the risk of falls and to guide rehabilitation.

Full-body reflective markers and walking mates are the reference tool for studying turning kinematics and stepping patterns in humans (15, 18, 20–25), but these tools are expensive and difficult to use in the clinics. Therefore, we used inertial measurement units (IMUs) for measurement, which are valid measuring tools for walking (26–28) and turning (29, 30) and are much cheaper and more compatible with routine use in clinical departments, retirement homes, and the patient’s home. Here, we studied the kinematics with IMUs of spontaneous 180° turns in poststroke left and right hemiparetic patients and controls and their relationship with risk of falls.

## Materials and Methods

### Participants

We included all stroke patients from the same physical and rehabilitation medicine department between July 2015 and October 2015; inclusion criteria were (a) only one stroke event leading to hemiparesis, (b) could perform a timed up-and-go test (TUG) and walk 10 m without human nor assistive aids, (c) giving informed consent, and (d) right-handed and right-footed for mobilization tasks according to the lateral preference questionnaire (Table A1 in Appendix) (31). Even if it has never been reported to our knowledge, “footedness” may affect the spontaneous turning side and the turning strategy (32). In fact, turning requires mobilization of an initiator limb contralateral to the first stance limb, which may be affected by footedness (33–35). We established a validated 8-item lateralization preference score ranging from −8 (consistent left) to +8 (consistent right); a score <4 and an answer “left” to the question “foot to kick a ball” excluded the participant.

We excluded patients with (a) multiple stroke, (b) a cerebellar syndrome, (c) a vestibular or musculoskeletal disorder that could affect walking or balance, and (d) an inability to understand the instructions. Patients were divided into RP and LP. The stroke type (ischemic or hemorrhagic) and location (hemispheric or pontine; see Table 1 for locations) were diagnosed by MRI. The etiology of the stroke had been established by adapted medical explorations. A poststroke period of ≤6 months at the time of the measurement was used to define chronic and recent strokes.

**Table 1:** **Characteristics of healthy controls (HCs) and poststroke patients who were left paretic (LP) or right paretic (RP)**.

		HC (*n* = 15)	LP (*n* = 20)	RP (*n* = 17)
Age (years), mean (SD)		56.7 (16.1)	60.7 (8.8)	57.5 (9.5)

Gender	Male	3	16	13
	Female	12	4	4

BMI (kg/m^2^), mean (SD)		25.3 (4.2)	25.3 (3.8)	26.4 (5.0)

Hemorrhagic stroke			5	9
Location	Cortico-subcortical hematoma		3	7
	Pontine hematoma		2	2
Etiology	Hypertension		5	3
	Others		2	4

Ischemic stroke			14	8
Location	Middle cerebral artery		13	8
	Anterior cerebral artery		1	0
Etiology	Atherosclerosis		8	3
	Arterial dissection		2	1
	Embolic heart disease		0	1
	Arteriovenous malformation		0	3
	Thrombocythemia		1	0
	Idiopathic		2	2

Time since stroke (months), mean (SD)			40.6 (49.2)	86.5 (133.4)

Strokes	Recent strokes (≤6 months)		4	5

	Chronic strokes (>6)		16	12
NFAC (/8)	≤5		7	9
	≥6		13	8
TUG (s), mean (SD)			16.4 (5.7)	19.1 (8.7)
FM (/34)	<20		3	3
	20–30		11	11
	>30		6	3

Sensitivity				
Proprioception(/2)	2		18	11
	1		2	5
	0		0	1
Thermal (/2)	2		19	15
	1		1	1
	0		0	1
Superficial (/1)	1		20	17
	0		0	0

Visuospatial neglect	No		17	17
	Yes		3	0

Homonymous hemianopsia	No		20	16
	Yes		0	1

Dysexecutive syndrome	No		18	16
	Yes		2	1

Aphasia	No		20	12

	Yes		0	5

Fallers (%)			15	29

*Data are no. unless indicated*.

*BMI, body mass index; FM, lower limb Fugl-Meyer scale; NFAC, new functional ambulatory categories; TUG, timed up-and-go test*.

*Sensitivity: 0 = anesthesia, 1 = normal (for superficial sensitivity) and hypoesthesia (for proprioception and thermal sensitivity), 2 = normal (for proprioception and thermal sensitivity), and fallers = 1 one-time fallers and repeat fallers*.

Functional ability was assessed in patients by the new functional ambulation categories (NFAC) test (36) (total score 8) and the TUG in seconds. Lower-limb motor control was assessed by the lower-limb Fugl-Meyer (FM) subscale (total score 34). We evaluated sensitivity *via* proprioceptive, thermal, and superficial deficits, graded 2 for no deficit, 1 for hypoesthesia, and 0 for anesthesia (superficial sensitivity deficit was graded 0 or 1); visuospatial neglect by the GEREN battery (37); visual field at the bedside; and dysexecutive syndrome and aphasia (graded present or absent).

The occurrence of falls within the 6 months after the measurement was assessed by a declarative fall questionnaire administered by phone call to patients or relatives. Patients were asked about the frequency, location, activity, and consequences of a fall, and whether they had injured themselves or experienced a fear of falling. Near-falls were not considered falls. Participants were classified as non-fallers (non-fallers with or without near-falls) or fallers (one-time fallers and repeat fallers) (9).

Healthy controls volunteered freely and had comparable age to patients. HC participants were right-handed and right-footed for mobilization tasks and had no neurological, vestibular, rheumatologic, or orthopedic disorders that could affect walking or balance.

This observational study was approved by the local ethics committee (Comité de Protection des Personnes Ile de France II, no. CPP 2014-10-04 RNI). Patients and controls gave their written informed consent to participate.

### Instrumentation and Data Acquisition

We measured linear accelerations and angular velocities of the lower back (L4–L5 vertebra) and the feet (dorsal face) by using three IMUs: triaxial accelerometers, gyroscopes, and magnetometers (Technoconcept, Mane, France; I4 Motion, autonomy 4 h Li-Ion battery, device dimensions 4.9 cm × 3.8 cm × 1.9 cm, acceleration range ±6 *g*, angular velocity range ±500°/s, sampling frequency 100 Hz, and angular velocity measurement error <1°/s). IMUs were attached to the body with manufacturer-designed adhesive straps and connected to a computer *via* Bluetooth (29). A linear drift correction was applied to the foot angular velocities by assuming null velocity of the foot during foot flat (FF) periods (38). A linear drift correction was applied to the axial angular position during the 180° turn by assuming 0° at the beginning of the turn and 180° when the turn was completed.

After sensor fixation, participants were instructed to execute the following task with their shoes on and without walking aids: stand quietly for 6 s at the starting point, walk at a comfortable walking speed to a previously shown turn point that was 10 m away (without previous specification of a turning side), and walk back to the starting point.

### 180° Turning Representation

**Figure 1:**
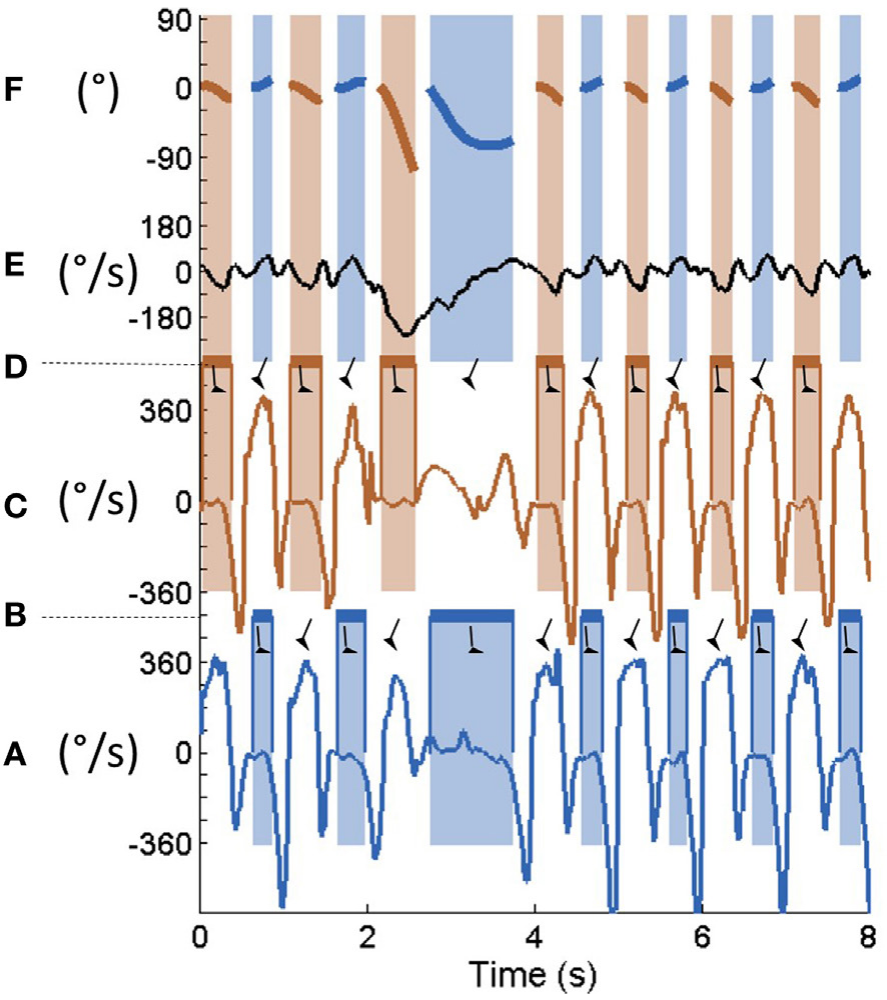
**Typical raw data for one healthy participant**. From the bottom to the top: right foot mediolateral angular velocity **(A)**. Right foot flat annotated from panel **(A)** is **(B)**. Left foot mediolateral angular velocity **(C)**. Left foot flat annotated from panel **(C)** is **(D)**. Lower back vertical angular velocity **(E)**. Lower back vertical angular position during each right (blue) and left (red) foot-flat obtained by integration of panel **(E)** during the colored time periods **(F)**. 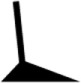 and 
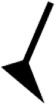
 represent foot-flat and swing phases, respectively.

**Figure 2:**
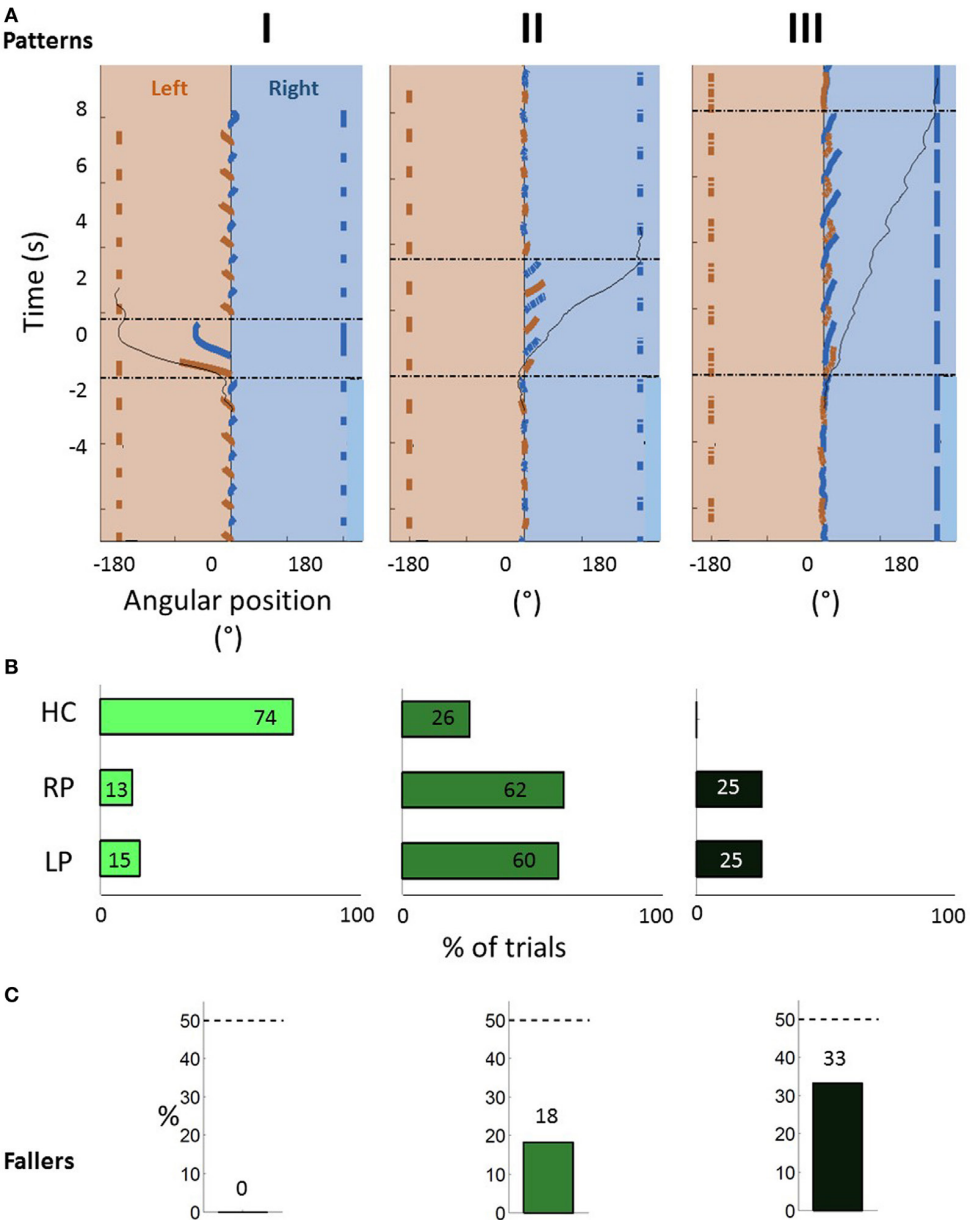
**(A)** Typical vertical angle covered by the trunk during each stance phase during walking and turning (see Figure 1 for details) for three participants presenting pattern I (1 external step), pattern II (2–4 external steps), and pattern III (≥5 external steps). Blue corresponds to the right side, and red background corresponds to the left side. Full lines at the left or at the right represent right (blue) and left (red) stance phases. Thin, dashed black lines indicate the start (time = 0 s) and the end of the turn. The thin continuous black curve corresponds to the cumulative angle during the 180° turn. **(B)** Spontaneous 180° turning patterns for healthy controls (HCs), right paretic (RP), and left paretic (LP) patients. Patterns I–III are represented by a gradient of green (from light to dark green). **(C)** Percentage of fallers among all paretic patients as a function of the spontaneous turning pattern.

Foot flat during walking and turning was manually annotated on the feet angular velocities in the sagittal plane (Figure 1) as previously validated by Mariani et al. (26). The rotation of the trunk in the axial plane during each FF period [i.e., axial rotation of the trunk during FF (ARTF)] was computed by integrating the angular velocity of the trunk in the axial plane during each FF period (Figure 1). The resulting data representation is shown in Figure 2A. Because ARTF never exceeded 10° during straight-ahead walking, a FF was considered part of a 180° turn when it exceeded a 10° empirical threshold, which allowed us to define both the 180° turn start and end.

### Turning Patterns

For each participant, the external number of steps, duration of the turn, and mean angular velocity of the trunk in the axial plane were computed. We defined three turning patterns (Figure 2A) according to the number of external steps taken. Pattern I involved 1 external step, pattern II from 2 to 4 steps and pattern III ≥5 steps.

### Turning Strategies

For each participant, we observed the spontaneous turn side and determined the stance limb during the first step of the turn (i.e., first stance limb), given by the 180° turning representation. According to an extension of previous literature that defined turning strategies for 90° turns in healthy populations (13, 19), we defined four turning strategies in our patients: strategy 1, turn toward the healthy side with a healthy first stance limb; strategy 2, turn toward the healthy side with a paretic first stance limb; strategy 3, turn toward the paretic side with a healthy first stance limb; and strategy 4, turn toward the paretic side with a paretic first stance limb.

### Statistical Analysis

The data were processed by using MATLAB^®^ (2013 version). Categorical variables were compared by chi-square test. Quantitative variables were compared by one-way ANOVA. Significance was defined as *p* < 0.05.

## Results

### Participants

We included 17 patients with RP [mean (SD) age 57.5 (9.5) years (range 43–73)], 20 with LP [mean age 60.7 (8.8) years (range 43–63)], and 15 HC [mean age 56.7 (16.1) years (range 36–83)]. RP and LP groups did not differ in age or clinical deficiencies and disabilities scored by FM, NFAC, TUG, and GEREN (Table 1). Five RP and four LP patients were recent strokes. All participants were right-handed, with a median score of 4 (range 4–8) on the lateralization scale and were right-footed for mobilization tasks. All participants performed the protocol.

### Turning Patterns

All 52 spontaneous 180° turns but one, which was erratic, were classified by turning patterns. Figure 2B shows the turning patterns in percentages for the HC, LP, and RP groups. The LP and RP groups behaved similarly in terms of turning patterns. Pattern III was absent in the HC group, and pattern I was unusual in the paretic groups (RP 12% and LP 15%). The mean (SD) turn duration significantly differed by turning patterns I, II, and III—2.3 (0.6), 3.8 (0.9), and 7.0 (3.3) s, respectively—as did the mean (SD) angular velocity of the trunk in the axial plane—80.0 (20.0), 52.3 (11.1), and 32.0 (0.7)°s, respectively. The mean (SD) TUG time differed by patterns II and III: 15.4 (3.8) and 25.7 (7.8) s. The NFAC and FM differed by turning patterns II and III and I and III (Table A2 in Appendix).

### Turning Strategies

**Figure 3:**
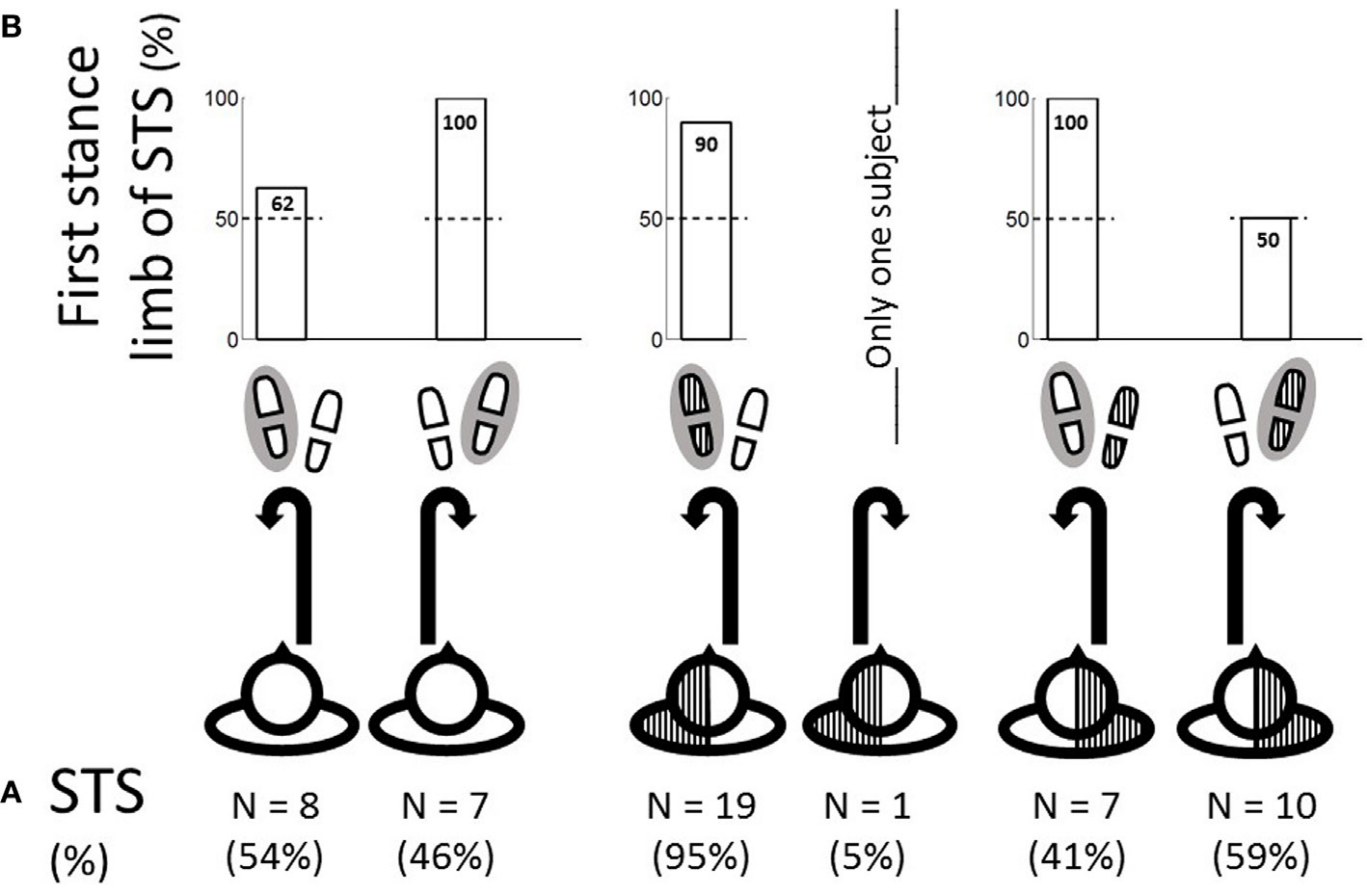
**(A)** Spontaneous 180° turn side (STS) for healthy controls, RP, and LP patients. The exercise is observed from the top. Hatched areas 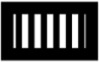 represent hemiparesis. **(B)** First stance limb (gray circle 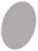) with respect to the spontaneous turn side (across the number *N* for each column).

The spontaneous 180° turn side for the groups is shown in Figure 3A: 54% of HC, 95% of LP, and 41% of RP patients chose the left side. The first stance limb by side of turn is in Figure 3B. Among HC subjects, for 62% of the participants who turned to the left and all participants who turned to the right, the first stance limb was the internal limb. Among LP and RP patients, for turns to the healthy side, the first stance limb was always the healthy and internal limb (strategy 1). For 90% of LP patients and 50% of RP patients who turned to the paretic side, the first stance limb was the paretic and internal limb (strategy 4). Globally, LP patients showed a marked predominance of strategy 4 (85%) as compared to RP patients (29%). No patient performed strategy 2.

**Figure 4:**
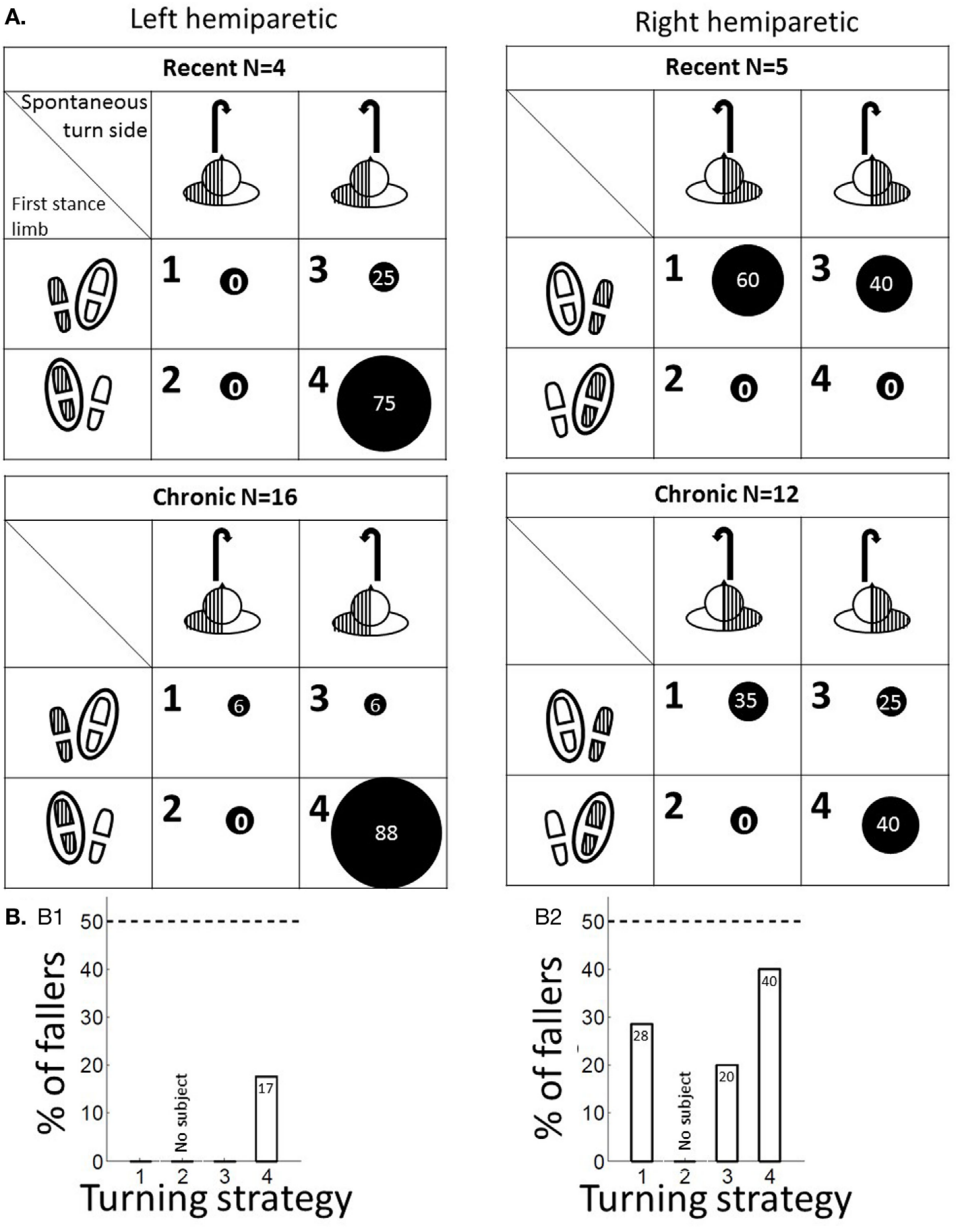
**(A)** Proportion of turning strategies for recent (time since stroke <6 months) and chronic (>6 months) LP and RP patients. Turning strategies numbered from 1 to 4 are indicated in the top left corner of each cell. The turning strategies are defined by the spontaneous turn side with respect to the hemiparesis side (scheme of the exercise observed from the top at the top of each column) and the stance limb during the first step of the turn (indicated by a black circle 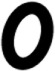 around the footprint at the left of each row). The two tables on the left show the data for LP patients, the two tables on the right show the data for RP patients, the two top tables show the data for recent strokes, and the two bottom tables show the data for chronic strokes. The proportion of patients in a given subgroup who performed a given turning strategy is represented by a proportional sized black circle. Hatched areas 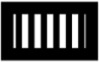 represent hemiparesis. **(B)** Proportion of fallers among LP (B-1) and RP patients (B-2) as a function of turning strategy.

The turning strategies by time since stroke are shown in Figure 4A. Among recent poststroke patients, none of the RP patients performed strategy 4 as compared with 75% of the LP patients. This percentage increased for chronic RP patients (40%) but remained constant for LP patients (88%).

Turning strategies were not associated with turning patterns. However, recent RP patients who performed strategy 1 had better turning kinematics, TUG, NFAC, and FM scores, than those who performed strategy 3 (Table A3 in Appendix). Chronic RP patients who performed strategy 1 or 3 had better TUG, NFAC, and FM scores than those who performed strategy 4. The corresponding data for LP patients are not shown because all but three performed strategy 4.

### Relation of Turning Strategy with Fall Incidence

During the 6 months after the turning measurement, 42 falls occurred in eight patients (four one-time fallers and four repeat fallers) (Table 2). The mean (SD) number of external steps required to perform the 180° turn was greater for fallers than non-fallers—4.0 (1.6) and 3.1 (0.7) (*p* = 0.026)—and mean (SD) TUG time was greater—23.1 (9.4) versus 16.3 (6.0) s (*p* = 0.018). The turn duration, mean angular velocity of the trunk in the axial plane, FM, and NFAC did not differ between non-fallers and fallers (Table A4 in Appendix).

**Table 2:** **Total number, circumstances, and consequences of falls**.

Total no. of patients	37
Total no. of fallers	8
Total no. of falls	42
Location	
Home (lounge or bedroom)	19
Home (toilet area)	5
Home (step or stairs)	8
Public area (public transportation)	6
Public area (street)	1
Public area (rehabilitation center)	3
Activity	
Walking	17
Turning	9
Transferring	10
Other	6
Injuries caused by falling	
No	38
Yes	4

*Injuries were two head traumas, one hip fracture, and one ankle injury*.

Fallers represented 29% of the RP patients and 15% of the LP patients. No patients who presented pattern I, 18% of patients who presented pattern II, and 33% of patients who presented pattern III were fallers (Figure 2C). The patient who was unclassified regarding turning patterns was a faller. In the RP and LP groups, the highest proportion of fallers were patients who performed turning strategy 4 (40 and 17%; Figure 4B). In the RP group, the proportion of fallers was greater among patients who performed strategy 1 or 4 (28 and 40%) than strategy 3 (20%). In the LP group, no patient who performed strategy 1 or 3 was a faller.

## Discussion

We investigated spontaneous 180° turns after 10 m of walking in a poststroke population with hemiparesis and controls by using three IMUs used for routine hospital practice. We described three turning patterns based on the number of external steps and extend the definitions of turning strategies to 180° turns in poststroke hemiparetic patients according to the spontaneous turn side and the first stance limb chosen. The LP and RP groups behaved similarly in turning patterns, but most LP patients chose to turn to the paretic side as compared with only about half of the RP patients. This difference increased with turning strategies: 85% of LP versus 29% of RP patients used strategy 4 (paretic turn side with paretic limb at first stance). The patients using strategy 4 had the highest rate of falls. Spontaneous turning strategies could be routinely assessed by using IMUs to identify risk of new falls among poststroke patients.

We found that IMUs are valid tools for locomotion measurements in stroke patients (27, 28). The positioning of three IMUs on the trunk and on the feet had been used for ambulatory monitoring of turns in Parkinson’s disease (30) and older-aged populations (39, 40), but this was the first time they were found appropriate to investigate poststroke turns in walking. IMUs appeared to perform adequately for defining the 180° turn start and end and for describing turning patterns and turning strategies in routine hospital practice. Actually, the stance limb can be recognized during the first turn step by only clinical observation. Usual turning factors (i.e., duration and mean axial angular velocity) could then be recorded also.

We considered participants who fell once as fallers based on previous results: for stroke patients, falls or near-falls while in hospital are strongly associated with falls when people return home (3, 4, 10) and older people in the general population who fall once are likely to become repeat fallers (3, 4, 41–43).

The spin and step turning strategies are commonly defined for 90° turns (12, 13, 17–19). Without the participant receiving specific instructions regarding turning, the reorientation toward the new direction of travel is often completed with more than one step (17). The step in the turn that should be chosen to define the turning strategy remains an open question (17, 19). In the Taylor et al. study, 20% of the turns were unclassifiable (13) and Fino et al. found variable results depending on the methods used to define turning strategies (19). For mechanical reasons, we hypothesized that the turn initiation constituted one critical instant for poststroke patients. Thus, we proposed the definition of 180° turning strategies considering the stance limb during the first step of the 180° turn. Strategies 1 and 4 are considered close to spin strategies and strategies 2 and 3 close to step strategies.

To our knowledge, spontaneous choice of turning has not been considered crucial information in previous studies imposing the side of turning (20, 21, 44, 45). Regarding falls in daily life, the side chosen for spontaneous turning may be important. In psychological studies of the spontaneous turn side in healthy subjects, 60% of right-handed participants turn to the left (46). With our conditions, 54% of right-handed HC participants turned spontaneously to the left. The consistency of our results with the literature validates our protocol and shows the absence of bias in the spontaneous choice of turning direction.

Turning patterns, as defined here, conveniently summarize the turning kinematics because they reflect the turning performances well (i.e., turn duration, number of external steps, and mean angular velocity) (11, 22, 47–49). Pattern I (<2 steps) was absent in paretic populations, which confirms the loss of pivoting during turning, as underlined by others (11, 22). The use of more steps when turning is thought to signify instability (39, 40, 48, 49). Suggestions for thresholds of number of steps to turn that indicate the risk of falls in community-dwelling older adults vary from 1 to >12 steps to complete a 360° turn (47) to ≥5 steps to accomplish a 180° turn (11, 22). Likewise, our findings show a relation between the number of steps taken to turn and risk of fall. In the only study to our knowledge that analyzed turning kinematics and risk of fall, turn duration and not number of steps differed between fallers and non-fallers (22). This discrepancy could be due to our analysis of spontaneous turns in daily clinical conditions. Hollands et al. imposed the turn side and performed the measurements in a gait analysis laboratory. Both these factors likely affect turning kinematics.

Considering turning strategies, turning toward the side of the stance limb reduces the polygon of lift (12, 13, 17–19). However, falls are more likely to occur during turns to the paretic side (9). Both the stance limb and the paretic turn direction could explain the highest rate of fallers among RP and LP patients (40 and 17%, respectively) among patients who performed strategy 4, a turn to the paretic side in the direction of the stance limb.

The LP and RP patients differed in turning strategies, with a marked predominance of strategy 4 among LP patients. This finding could suggest a differential role of both hemispheres in postural control (50). Anosognosia, neglect, and dysexecutive syndrome are predominant in right-hemispheric lesions of right-handed people (51) (i.e., among our LP patients, except for two who had a subcortical pontine stroke). We believe that turning strategy 4 is more natural because turns to the paretic side are simpler for mechanical reasons (15, 20, 21). Furthermore, our results among healthy subjects suggest that initiating the 180° by standing on the internal limb is more natural. Thus, mechanical easiness and a lack of consciousness of danger could explain why LP patients spontaneously adopt strategy 4.

Among RP patients, a good consciousness of the danger may lead to a modulation of turning strategies. Among recent RP patients, those with good motor skills turned to the healthy side, following the natural tendency of healthy participants. However, recent RP patients with high motor impairment turned to the paretic side for mechanical easiness, but they modulated the turning strategy by standing on the healthy external limb at turn initiation. By doing so, they adopted a safe behavior. Of note, chronic RP patients with poor turning performance adopted strategy 4. They may have adopted that risky and natural behavior as they became accustomed to their disability.

Even if stroke location is debated as a risk factor of falling, falls are considered more frequent in LP than RP patients (9). In the present study, falls were more frequent in RP than LP patients (29 and 15%, respectively). This finding could be explained by the declarative fall questionnaire in that patients with right hemisphere lesions tend to minimize fall occurrence.

“Footedness for the mobilization tasks” designates the lower limb that is spontaneously used in asymmetric lower-limb tasks requiring active mobilization (such as kicking a ball) as opposed to stabilization tasks (standing on one foot) (33–35). Turning requires mobilization of an initiator limb contralateral to the first stance limb (33–35). Previous findings in right-footed subjects show consistency and strong preference for the right foot in mobilization tasks as compared with a weak-foot preference in stabilization tasks in right-lateralized populations (33–35). Thus, footedness may affect the spontaneous turning strategy in the choice of the initiator limb (i.e., foot contralateral to the first stance limb) (32). In the present study, all control and poststroke subjects were right-footed for mobilization tasks. Only 33.5% of the control subjects spontaneously choose to turn left with the right limb as initiator. Thus, the coincidence between footedness for mobilization tasks and initiating turning is not found in 66.5% of the subjects turning right with the left limb as initiator, which suggests that other aspects are at stake (32). Recent and chronic LP and recent RP stroke survivors prefer to use the dominant right limb as the initiator limb (contralateral to the first stance limb) with strong consistency regardless of behavioral context (side of spontaneous turn and side of hemiparesis) (33). When the dominant right limb is paretic, then over time (chronically >6 months), the left leg may become dominant and increasingly used as the initiator limb (Figure 4). Bonifer et al showed that for stroke patients, the paretic arm (52, 53), even when dominant before stroke, only assisted the healthy arm after stroke. For the lower limb, we can only infer an analogous trend. However, the difference of proportion in turning strategies between HC and stroke patients implies that mechanical easiness and neglect may also play a determinant role in the choice of turning strategy.

Fall risk is due to multiple factors. Our study proposes considering turning strategies according to the side of the hemiparesis as new indicators to evaluate the risk of fall after stroke. The question of the importance of this aspect among other well-known risk factors [balance impairment (2–4, 43, 54) with sensitive deficits, motor deficits, and hemineglect (1, 55–57)] is of interest for future study. The spontaneous choice of turning and the number of external steps taken do not require the use of IMUs in daily clinical practice. Yet, to identify the first stance limb of the 180° turn, IMUs can give better information with minimal need for space. In this approach, light IMU instrumentation could also become an interesting extension of clinical examination to teach patients secured turning strategies in a rehabilitation setting. Turning strategies evaluated by IMUs can be proposed to provide customized balance rehabilitation programs.

## Ethics Statement

This observational study was approved by the local ethics committee (Comité de Protection des Personnes Ile de France II, no. CPP 2014-10-04 RNI). Patients and controls gave their written consent to participate.

## Author Contributions

Conceptualization and methodology: AY, SB, P-PV, and DR. Software: RB, LO, and AV. Validation: RB, AV, LO, and DR. Formal analysis: RB, LO, AY, P-PV, AV, and DR. Investigation: RB, AY, DR, LT, and CP. Resources: AY, LT, and CP. Data curation: RB. Writing (original draft preparation): RB, LO, DR, and AY. Writing (review and editing): RB, LO, DR, AY, and P-PV. Visualization: RB and DR. Supervision: AY, P-PV, and DR. Project administration: RB, AY, P-PV, and DR. Funding acquisition: P-PV.

## Conflict of Interest Statement

The authors declare that the research was conducted in the absence of any commercial or financial relationships that could be construed as a potential conflict of interest.

## Abbreviations

IMU, inertial measurement unit; HC, healthy control; LP, left paretic; RP, right paretic; FF, foot flat; ARTF, axial rotation of the trunk during foot flat.

## Appendix

**Table A1:** **Lateral preference questionnaire**.

Questions	Right	Both	Left
With which hand do you write?			
With which hand do you hold a toothbrush?			
With which hand do you use a hammer?			
With which hand do you throw a ball to hit a target?			
With which foot do you kick a ball?			
If you went to step on one leg, which foot would you step on?			
Which eye do you use when looking through a telescope?			
Which ear do you use to listen to the telephone?			

Each item was scored −1 for “left,” 0 for “both”, and 1 for “right.”

*Participants were considered right-lateralized with score >3*.

**Table A2:** **Characteristics of turning patterns (pattern I = 1; II = 2–4; III ≥ 5 external steps)**.

		Turning pattern			
		I	II	III	
**For all participants**		*n* = 16	*n* = 26	*n* = 9	
No. of external steps, mean (SD)		1.0 (0.0)	2.9 (0.6)	5.3 (1.8)	NA
Turn duration (s), mean (SD)		2.3 (0.6)	3.8 (0.9)	7.0 (3.3)	*
Angular velocity (°/s), mean (SD)		80.0 (20.0)	52.3 (11.1)	32.0 (0.7)	*
**For patients only**		*n* = 5	*n* = 22	*n* = 9
Timed up-and-go test (s), mean (SD)		11.3 (2.7)	15.4 (3.8)	25.7 (7.8)	II–III
New functional ambulation categories (/8)	≤5	20 (1)	32 (7)	77 (7)	I–III
	≥6	80 (4)	68 (15)	23 (2)	II–III
Lower-limb Fugl-Meyer subscale (/34)	<20	0	0	55 (5)	I–III
	20–30	60 (3)	68 (15)	45 (4)	II–III
	>30	40 (2)	32 (7)	0	

*Data are no. (%) unless indicated*.

**Significant difference between all the columns and II–III = significant difference between two columns (*p* < 0.05)*.

*NA, not applicable for qualitative parameters*.

*One turn could not be classified as any pattern*.

**Table A3:** **Characteristics of turning strategies in right paretic (RP) patients**.

		Turning strategy			
		1	2	3	4
**Recent RP**		*n*−3	*n* = 0	*n*−2	*N* = 0
No. of external steps, mean (SD)		2.7 (0.6)	–	3.5 (0.7)	–
Turn duration (s), mean (SD)		3.3 (0.7)	–	4.9 (0.4)	–
Angular velocity (°/s), mean (SD)		62.7 (4.5)	–	38.0 (0.7)	–
Timed up-and-go test (TUG) (s), mean (SD)		10.8 (0.4)	–	19.5 (0.6)	–
New functional ambulation categories (NFAC) (/8)	≤5	33 (1)	–	100 (2)	–
	≥6	66 (2)	–	0	–
Lower-limb Fugl-Meyer subscale (FM) (/34)	<20	0	–	0	–
	20–30	33 (1)	–	100 (2)	–
	>30	66 (2)	–	0	–
Turning pattern	I	0	–	0	–
	II	100 (3)	–	100 (2)	–
	III	0	–	0	–
**Chronic RP**		*n* = 4	*n* = 0	*n* = 3	*n* = 5
No. of external steps, mean (SD)		3.5 (1.3)	–	3.7 (0.6)	3.4 (1.1)
Turn duration (s), mean (SD)		3.9 (1.0)	–	5.2 (1.0)	6.2 (2.7)
Angular velocity (°/s), mean (SD)		55.5 (13.3)	–	36.9 (7.1)	36.6 (16.5)
TUG (s), mean (SD)		16.4 (5.9)	–	16.6 (4.8)	27.4 (10.7)
NFAC (/8)	≤5	25 (1)	–	33 (1)	80 (4)
	≥6	75 (3)	–	66 (2)	20 (1)
FM (/34)	<20	25 (1)	–	0	40 (2)
	20–30	75 (3)	–	66 (2)	60 (3)
	>30	0	–	33 (1)	0
Turning pattern	I	25 (1)	–	0	25 (1)
	II	25 (1)	–	100 (3)	25 (1)
	III	50 (2)	–	0	50 (2)

*Data are no. (%) unless indicated*.

*The data for left paretic patients are not shown because 85% performed strategy 4*.

*Significant difference between all the columns and 1–4 = significant difference between two columns (*p* < 0.05)*.

*NA, not applicable for qualitative parameters*.

*One turn could not be classified as any pattern*.

**Table A4:** **Characteristics of fallers**.

		Non-fallers	Fallers	
Size		29	8	
No. of external steps, mean (SD)		3.1 (0.7)	4 (1.6)	*
Turn duration (s), mean (SD)		4.2 (1.5)	5.3 (1.9)	
Angular velocity (°/s), mean (SD)		49.7 (15.3)	42.2 (14.5)	
Timed up-and-go test (s), mean (SD)		16.3 (6.0)	23.1 (9.4)	*
New functional ambulation categories (/8)	≤5	41 (12)	50 (4)	
	≥6	58 (17)	50 (4)	
Lower-limb Fugl-Meyer subscale (/34)	<20	10 (3)	37 (3)	
	20–30	62 (18)	50 (4)	
	>30	27 (8)	12 (1)	

*Data are no. (%) unless indicated*.

**Significant difference (*p* < 0.05)*.

*Two trials from the same subject could not be classified in any pattern*.

*NA, not applicable for qualitative parameters*.

*One patient could not be classified in turning patterns*.
